# Investigation of the Mechanical Properties of the Human Tracheal Cartilage

**Published:** 2017

**Authors:** Farzaneh Safshekan, Mohammad Tafazzoli-Shadpour, Majid Abdouss, Mohammad Behgam Shadmehr, Fariba Ghorbani

**Affiliations:** 1 Faculty of Biomedical Engineering, Amirkabir University of Technology, Tehran, Iran,; 2 Chemistry Department, Amirkabir University of Technology, Tehran, Iran,; 3 Tracheal Diseases Research Center, National Research Institute of Tuberculosis and Lung Diseases (NRITLD), Shahid Beheshti University of Medical Sciences, Tehran, Iran.

**Keywords:** Trachea, Airway, Cartilage, Mechanics

## Abstract

**Background:**

The tracheal cartilage plays an important role in maintaining the mechanical stability of the trachea, as it keeps the trachea open and prevents its collapse under the negative pressures of the respiratory cycle. This study aimed to evaluate and compare the mechanical properties of cartilage specimens from the cranial and caudal regions of the human trachea and compare the results with respect to age and sex of the subjects.

**Materials and Methods:**

After obtaining human trachea samples from brain-dead, organ-donating patients and storing them in appropriate conditions, the prepared cartilage samples from the cranial and caudal regions of the trachea were subjected to uniaxial tension and stress relaxation experiments to obtain the corresponding Young’s modulus and relaxation percentage values, respectively. The results were compared in terms of the position (cranial or caudal) in the trachea, and age and sex of the patients.

**Results:**

Based on the results, no statistically significant effect of the position in the trachea on the Young’s modulus of the human tracheal cartilage samples was observed, despite the generally stiffer behavior of cartilage samples from the cranial region compared to those from the caudal region of the trachea. For both the cranial and caudal regions, no significant effect of sex on the stiffness of the tracheal cartilage was observed; further, the cartilage samples of the human trachea (from both cranial and caudal regions) of the old subjects were significantly stiffer than those of the young subjects. Based on the stress relaxation data, no significant effect of age, sex, or position on the relaxation percentage was observed.

**Conclusion:**

The tracheal cartilage samples of the old patients are significantly stiffer than those of the young patients. Sex and position in the trachea (cranial vs caudal) do not significantly influence the mechanical properties of the human tracheal cartilage samples. The results of this study can be useful in designing tracheal tissue-engineered scaffolds, which should be mechanically compatible with the native trachea.

## INTRODUCTION

The trachea is a unique part of the conducting airways, and it consists of 18 to 22 distinct C-shaped cartilaginous rings. These rings are completed by smooth muscles in the posterior region of the trachea, and the spaces between them are composed of connective tissue (1,2).

The tracheal cartilage plays a crucial role in the mechanical function of the trachea, which directly affects its physiological respiratory function. The cartilaginous parts are the stiffest tracheal constituents and they keep the lumen of the trachea open even during negative pressures, hence inhibiting tracheal collapse and air flow limitation (3).

As a result of different pathological conditions, the stiffness of the tracheal wall is weakened or the free diameter of the lumen for air flow is reduced; breathing problems can therefore occur (4). Tracheal tissue engineering proposes to treat an injured trachea using cells and scaffolds, which should mechanically resemble the native trachea, and thus be able to function appropriately under normal respiratory conditions in vivo (5). Therefore, a proper knowledge of the mechanical properties of the native tracheal tissues, particularly the tracheal cartilage, is of great importance for the future success of tracheal tissue engineering.

The elastic behavior of the tracheal cartilage has been previously assessed and it has been considered as a linear (3,6, 7) or non-linear (2,4, 8,9–12) elastic material. The stress-strain behavior of the cartilaginous rings of a single trachea has been reported to vary along its longitudinal axis (10), a finding that was limited by the insufficient number of the studied subjects. In addition, the simulated collapsibility of the trachea has been reported to differ in the cranial and caudal regions of the trachea as a result of the different geometries of the cartilage rings in such positions (6,13).

Previous studies on the viscoelastic properties of the human trachea have either considered this organ in general to examine the time-dependent “tube law” of the trachea (pressure-volume relationship) (14,15) or have focused on the tracheal mucus (16–18). However, to our knowledge, the viscoelastic behavior of the human tracheal cartilage has not been well studied.

The cranial and caudal parts of the trachea differ in their anatomical and mechanical conditions. The cranial region of the trachea, which is close to the cricoid cartilage and the inferior margin of the larynx, is outside the chest space; therefore, it does not experience the existing negative pressure. Conversely, the caudal part that rests above the tracheal bifurcation is subjected to the negative pressure in the chest (19).

In the major bronchi, the cartilage is still present, although in a more random organization. The cartilaginous content of the tracheal wall further decreases downwards, such that in the peripheral airways with diameters of approximately 1 mm, cartilaginous regions are no longer present (20).

To assess the variation of the mechanical properties of the tracheal cartilage along the longitudinal axis of the trachea better, this study aimed at comparing the elastic and relaxation behaviors of cartilage samples from the cranial and caudal regions of the human trachea and the results in terms of sex and age of the subjects.

## MATERIALS AND METHODS

In the present study, tracheal cartilage specimens from the cranial and caudal regions of the trachea were prepared and exposed to uniaxial tension or stress relaxation tests to obtain stress-strain or stress-time data and subsequently calculate the Young’s modulus and relaxation percentage values, respectively, after isolating the samples of the human trachea from brain-dead and organ-donating patients and storing them in suitable conditions. The results were compared in terms of the position (cranial or caudal) in the trachea and age and sex of the subjects (13 young subjects: eight men and five women; and 17 old subjects: 10 men and seven women). The mean age of the young (aged 18 to 36 years) and old (aged 49 to 65 years) subjects was 26.54±6.45 and 55.88±6.29 years, respectively.

### Sample preparation

The present study was approved by the Ethics Committee of Masih Daneshvari Hospital (ethics code of sbmu1.rec.1393.73). After obtaining informed consent from the family members of the brain-dead patients, 30 samples of human trachea were isolated from these organ-donating patients, who had with no airway problem. Each isolated tracheal sample was immediately transferred to a container with a physiologic saline solution (as an appropriate preservation solution (21)), and then stored at −20°C to minimize the risk of tissue deterioration (7,10, 22). Each trachea was thawed before mechanical testing by placing in the refrigerator at 4°C, 24 hours prior to the experiments, and in an ambient temperature, 3 hours before the mechanical tests. The tracheal cartilage samples were cut into desired dimensions for testing. To minimize the effects of curvature on the results and provide uniaxial tension conditions, only the central and straight region of each cartilaginous sample was utilized for the experiments (10). The average initial length (L_0_) of the tracheal cartilage specimens was 3.87±1.69 mm.

### Mechanical tests

Mechanical tests on the human tracheal cartilage samples were performed at room temperature using a Zwick/Roell testing machine (Zwick/Roell GmbH, Ulm, Germany). Sandpaper pieces were placed between the specimen and the fixtures to prevent sliding of the specimen during the tensile test.

Each cartilage specimen was first subjected to a preconditioning step (6 loading/unloading cycles with a strain rate of 0.1 mm/min and a maximum strain level of 1%) to obtain repeatable results (2) and uniaxial tension thereafter (with a 0.5 mm/min strain rate until sample failure or stress relaxation by applying a constant strain amplitude of 10% on the sample and then allowing it to relax over 300 s). A physiologic saline solution was used to moisturize the specimens during mechanical testing.

In the tensile tests, the force displacement data recorded by the tensile machine were converted to stress-strain data using the following equations:

Equation 1 $$\varepsilon =\frac{\Delta L}{L_{0}}$$

Equation 2 $$\sigma =\frac{F}{A_{0}}$$

where ΔL and F represent the displacement and tensile force, respectively; σ represents stress; ε represents strain; and finally, A_0_ and L_0_ represent the initial cross section area and length of the specimen, respectively.

The Young’s modulus (E), also known as elastic modulus, which defines the relationship between stress (σ) and strain (ε) in a linear elastic material, can be calculated using the following equation:

Equation 3 $$E=\frac{\sigma }{\varepsilon }$$

For the stress relaxation experiments, the force-time data obtained were converted to stress-time data using Equation 2. Thereafter, the relaxation percentage of each specimen was calculated using the following equation:

Equation 4 $$\mathrm{Rel}\%=(\sigma_{\mathrm{initial}-}\sigma_{\mathrm{equilibrium}})/\sigma_{\mathrm{initial}}$$

where Rel% represents the relaxation percentage; σ_initial_ represents the initial stress magnitude in response to the application of the constant strain of 10% on the sample at the beginning of the relaxation test; and σ_equilibrium_ represents the final and equilibrium stress level after relaxation over 300 s.

### Statistical analysis

All statistical analyses were performed using the SPSS 16.0 software. To compare the results, the normal distribution of the data was first checked using the Shapiro-Wilk test, which resulted in P values greater than 0.05 (The null-hypothesis of this test is that the population is normally distributed).

Thereafter, the *t*-test and three-factor ANOVA analysis were performed to compare the experimental results obtained from the tracheal cartilage samples with respect to the position in the trachea (cranial vs caudal), and age and sex of the subjects, with the significance level set at 0.05. All data were presented as means±SDs.

## RESULTS

### Mechanical tests

**Figure 1.**
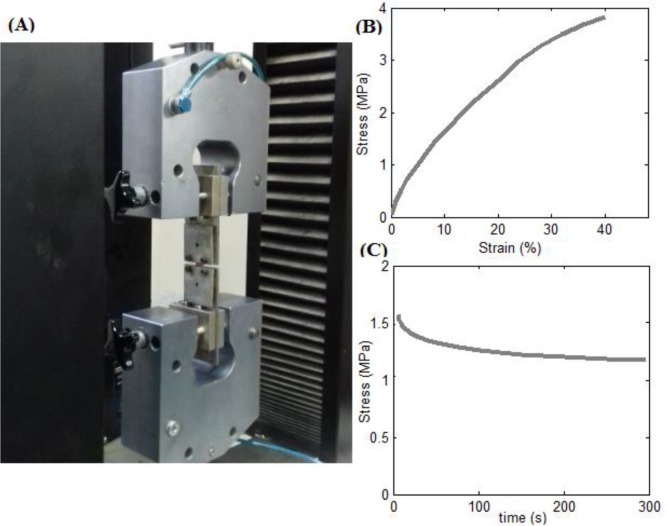
(A) A sample of human tracheal cartilage under mechanical testing, and typical (B) stress-strain and (C) stress-time curves for tracheal cartilage sample of a 36 year-old male subject.

Using Equations 1 and 2, the force-displacement and force-time data from the tensile and stress relaxation experiments were converted to stress-strain and stress-time data, respectively. Figure 1 illustrates a cartilage sample subjected to mechanical testing along with typical stress-strain and stress-time curves obtained for the tracheal cartilage of a 36-year-old subject.

Although the majority of the cartilage samples could experience strains up to 40% to 50% (as illustrated in Figure 1 for a typical sample), with the aim of taking all samples into account, averaging was conducted until a strain of 20% was reached, which fully covers the physiologic range of tracheal cartilage deformation (less than 5% of strain (23)).

### Effects of position, age, and sex on the tracheal cartilage mechanics

#### Stress-strain data

After verification of the normality of data distribution, the three-way ANOVA was used to assess the effects of position in the trachea, sex, and age on the stiffness and relaxation percentage of the cartilage samples. Based on the results, there was no statistically significant three-way interaction among position, age, and sex (P=0.636, F=0.238). Furthermore, there was no significant two-way interaction among the different pairs of factors (age×sex: F=0.123, P=0.733; age×position: F=0.080, P=0.989; sex×position: F=0.067, P=0.801).

**Figure 2.**
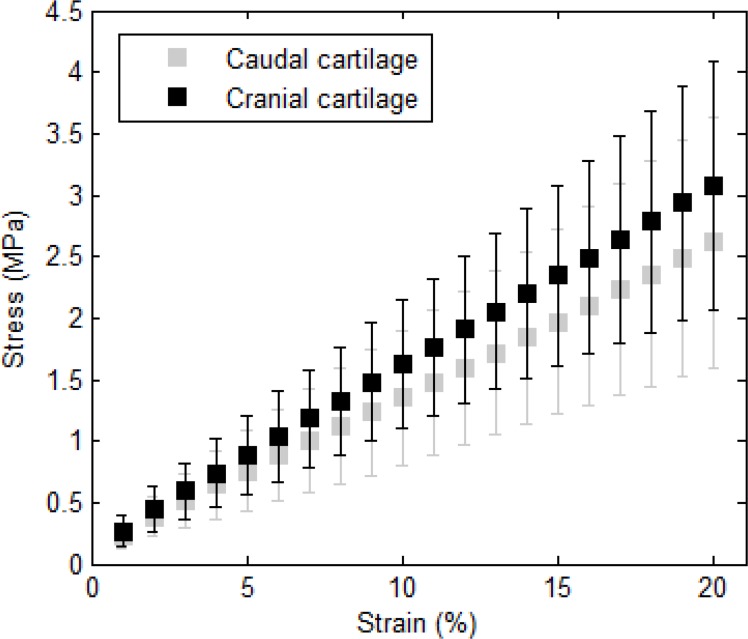
Average stress-strain curves corresponding to the cartilaginous samples from the cranial and caudal regions of the human trachea. No significant difference between the two groups is observed (P=0.146).

With respect to position in the trachea, Figure 2 illustrates the average curves corresponding to the cartilaginous samples from the cranial and caudal regions of the human trachea. Despite the generally stiffer behavior of the cartilage samples from the cranial region than that from the caudal part of the trachea, no statistically significant difference was observed (P=0.146); hence, the position of the trachea did not have a significant effect on the Young’s modulus of the human tracheal cartilage samples (Table 1).

**Table 1.** The results of Young’s modulus values for tracheal cartilage, in terms of age, gender and position in trachea (cranial vs. caudal).

**Factor**	**Group**	**Young’s modulus**	**F value**	**P value**
**Age**	Young	12.178±1.265	0.022	0.004
	Old	20.540±1.840		
**Gender**	Female	16.227±1.623	0.084	0.908
	Male	16.491±1.534		
**Position**	Cranial	18.119±1.538	2.486	0.146
	Caudal	14.597±1.692		

**Figure 3.**
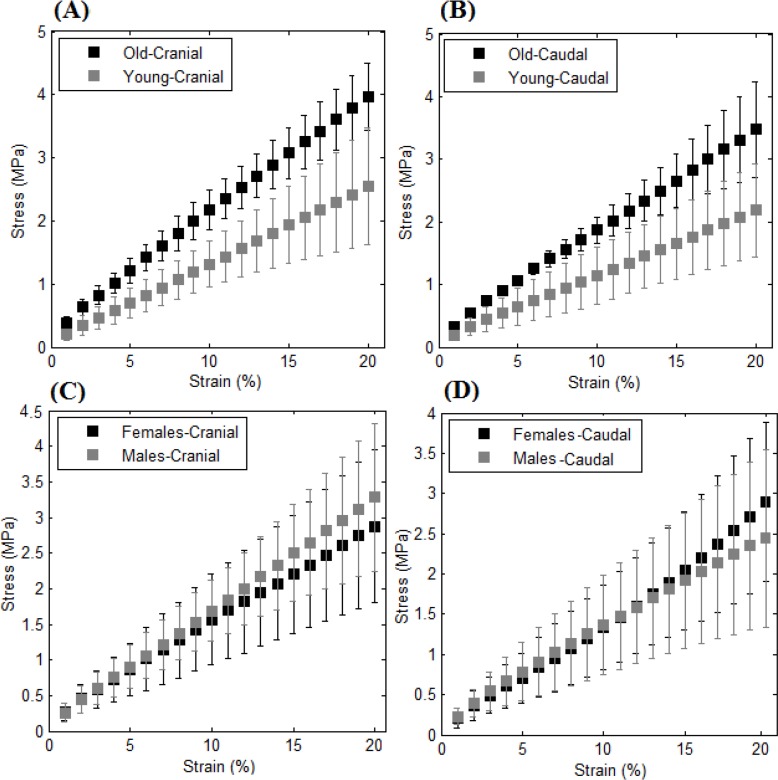
Average stress-strain curves of the human tracheal cartilage: (A) age-based comparison for the cranial region (F=7.878, P=0.031); (B) age-based comparison for the caudal region (F=6.102, P=0.049); (C) sex-based comparison for the cranial region (F=0.80, P=0.786); and (D) sex-based comparison for the caudal region (F=0.008, P=0.931).

The stress-strain curves corresponding to the cranial and caudal regions of the trachea are compared in terms of age and sex as illustrated in Figure 3. As shown in this figure, no significant effect of sex on the stiffness of the tracheal cartilage is observed (P=0.908); however, the cartilage samples of the human trachea from the old subjects are significantly stiffer than those from the young subjects (P=0.004) for both the cranial and caudal regions.

**Figure 4.**
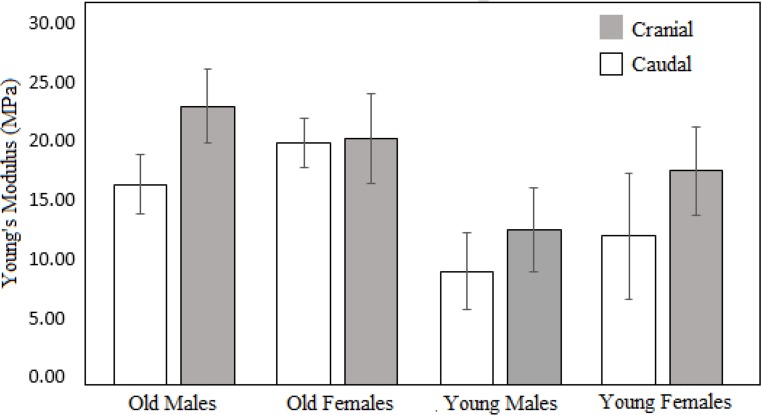
Average values corresponding to Young’s modulus of tracheal cartilage samples based on position in trachea (cranial vs. caudal), age and gender.

Until 10% of strain, there were almost linear trends of the stress-strain curves; therefore, the data were used to calculate the corresponding Young’s modulus values for all samples. The average Young’s moduli, corresponding to the different groups of tracheal cartilage samples, are illustrated in Figure **4**.

The results of age-, sex-, and position-based comparisons of the groups along with the corresponding P values are listed in Table 1.

As evident in Figure 4 and Table 1, we noticed a significant effect of age on the mechanical properties of the tracheal cartilage samples (F=0.022, P=0.004). The elastic moduli corresponding to the cartilage samples of the old subjects were significantly greater than those of the young subjects. However, there was no statistically significant effect of sex on the mechanical properties of the tracheal cartilage samples (F=0.14, P=0.908).

### Stress relaxation data

Using Equations 2 and 4, the force-time data from the stress relaxation experiments were converted to stress-time data, and the corresponding relaxation percentage of each sample, which is a good measure for comparing the relaxation behaviors of different specimens, was calculated. Figure 1(C) shows a typical stress relaxation curve for the tracheal cartilage of a 36-year-old patient.

Following verification of the normal distribution of the data, we used the three-way ANOVA to investigate the effects of position, sex, and age on the relaxation percentage of the cartilage samples. Based on the results, no statistically significant three-way interaction among position, age, and sex was observed (F=3.750, P=0.112). Furthermore, there was no significant two-way interaction among the different pairs of factors (age×sex, age×position, and sex×position had the following values: F=0.730, P=0.415; F=0.132, P=0.735; and F=2.096, P=0.221, respectively).

**Figure 5.**
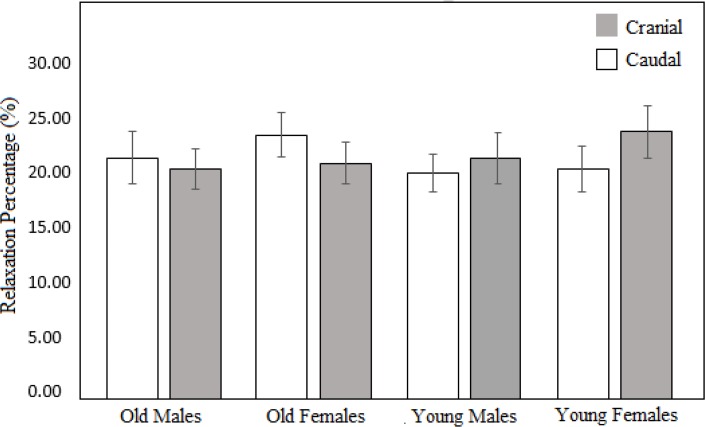
Average values corresponding to relaxation percentage of tracheal cartilage samples based on position in trachea (cranial vs. caudal), age and gender.

The results obtained for the relaxation percentage in terms of age, sex, and position of the samples are shown in Figure 5 and Table 2.

**Table 2.** Comparison of the relaxation percentage values for tracheal cartilage, in terms of age, gender and position in trachea (cranial vs. caudal).

**Factor**	**Group**	**Relaxation percentage (%)**	**F value**	**P value**
**Age**	Young	22.71±4.12	7.002	0.061
	Old	24.13±7.11		
**Gender**	Female	22.67±5.15	6.944	0.069
	Male	24.01±3.65		
**Position**	Cranial	23.45±6.17	0.301	0.612
	Caudal	23.06±5.34		

As evident in Figure 5 and Table 1, we noticed no significant effect of age, sex, or position on the relaxation behavior of the human tracheal cartilage samples.

## DISCUSSION

In the present study, the stress-strain data and stress relaxation behavior of the cartilage samples from the cranial and caudal regions of the human trachea were assessed. To the best of our knowledge, such a position-based comparison of the mechanical behavior of the tracheal cartilage using a reasonably large population of samples has not been previously performed. The number of subjects studied here was sufficient to allow comparison of the results with respect to the age and sex of the subjects.

Histologically, the tracheal cartilage is of a hyaline type, with an extracellular matrix composed mainly of collagen type II. In previous studies, the tracheal cartilage has been considered as a linear (3,6, 7) or non-linear (2,4,8–12) elastic tissue. Previous studies on the viscoelastic properties of the human trachea have examined the time-dependent “tube law” of the whole trachea (i.e., pressure-volume relationship) (14,15) or only the tracheal mucus (16–18). However, to our knowledge, the viscoelastic behavior of the human tracheal cartilage has not been well studied.

It has been previously reported that the collapsibility of the trachea in different tracheal regions (i.e., cranial, median, and caudal) is different; the caudal region is the most compliant part of the trachea (6,13). Such findings have been explained by the different geometries of the cartilaginous rings along the tracheal longitudinal axis. The tips of the C-shaped cartilages are closer to each other in the cranial region than in the caudal region of the trachea, where the tips are farther from each other and have been considered to facilitate tracheal deformation and collapsibility. In another study (10), different cartilaginous rings of a single trachea sample have been reported to exhibit relatively different stress-strain behaviors; however, the results were limited by the number of studied samples.

Considering the different physiological aspects of the cranial and caudal regions of the trachea, we compared the mechanical properties of the tracheal cartilage from the cranial and caudal regions. In the present study, we calculated and compared the Young’s modulus and relaxation percentage values of different groups of specimens (in terms of position in the trachea, age, and sex).

Based on our results, the cartilage specimens from the cranial region were generally, but not significantly, stiffer than those from the caudal region of the human trachea. Therefore, no significant effect of position in the trachea on the mechanical properties was observed.

We compared the Young’s modulus and relaxation percentage results obtained from the samples in the two positions in the trachea and observed similar age- and sex-related mechanical behaviors for both the cranial and caudal samples.

In both groups, no significant effect of sex on the mechanical behavior of the trachea samples was observed. However, age significantly influenced the stress-strain behavior of the tracheal cartilage samples in both groups. In fact, the cartilage specimens of the old subjects were significantly stiffer than those of the young subjects for both the cranial and caudal regions of the human trachea. This finding is in agreement with the previously reported increase in the stiffness of the tracheal cartilage with aging (7).

We observed no significant effect of age, position, and sex on the relaxation percentage of the tracheal cartilage samples, which is in contrast to the result of a previous study on the costal cartilage, where more relaxation of this tissue has been observed in the male subjects than in the female subjects (24).

In this study, the tissues were harvested from brain-dead patients. Thus, there were some limitations owing to tracheal intubation, including intubation duration, risk of tissue degeneration, and subsequent fibrosis and tracheal stenosis (25), and also owing to the possibility of local infection and underlying medical conditions, such as diabetes or musculoskeletal problems (8).

Regardless of the genetic factors, all these confounding variables were managed via case selection.

The intubation duration was 7 to 12 days for all donors. Underlying diseases and local infection were ruled out during the assessment for organ donation. In all cases, the cause of brain death was head trauma.

In previous studies, other influential factors, such as exercise and nutritional status, have been considered in the articular cartilage (26,27). However, the impacts of such factors have not been studied in the tracheal cartilage. Furthermore, the characteristics of inhaled air, including pollution or atmospheric pressure, may also affect the mechanical characteristics of the tracheal cartilage.

In the present study, we tested and compared the mechanical behavior of the cartilage samples from the cranial and caudal regions of the human trachea. The cartilage samples from the cranial region of the trachea were generally, but not significantly, stiffer than those from the caudal region. We also assessed the effects of age and sex on the results. In both the cranial and caudal samples, the cartilage specimens of the older subjects were significantly stiffer than those of the younger subjects. However, no significant effect of sex on the mechanical behavior of the tracheal cartilage was observed. Further, no significant effect of age, position, and sex on the relaxation percentage of the tracheal cartilage samples was observed. The results of this study can improve our understanding of the mechanical properties of the human trachea and can be employed in designing scaffolds for tracheal tissue engineering.
